# Experimental determination of the respiratory tract deposition of diesel combustion particles in patients with chronic obstructive pulmonary disease

**DOI:** 10.1186/1743-8977-9-30

**Published:** 2012-07-28

**Authors:** Jakob Löndahl, Erik Swietlicki, Jenny Rissler, Agneta Bengtsson, Christoffer Boman, Anders Blomberg, Thomas Sandström

**Affiliations:** 1 Department of Physics, Division of Nuclear Physics, Lund University, P.O. Box 118, 221 00, Lund, Sweden; 2 Department of Design Sciences, Division of Ergonomics and Aerosol Technology (EAT), Lund University, P.O. Box 118, 221 00, Lund, Sweden; 3 Energy Technology and Thermal Process Chemistry, Umeå University, 901 87, Umeå, Sweden; 4 Department of Public Health and Clinical Medicine, Division of Medicine/Respiratory Medicine, Umeå University, 901 87, Umeå, Sweden

**Keywords:** Lung deposition, Toxicity, Health effects, Air pollution, Agglomerate, Nanoparticles, Aerosol, COPD, Diesel exhaust

## Abstract

**Background:**

Air pollution, mainly from combustion, is one of the leading global health risk factors. A susceptible group is the more than 200 million people worldwide suffering from chronic obstructive pulmonary disease (COPD). There are few data on lung deposition of airborne particles in patients with COPD and none for combustion particles.

**Objectives:**

To determine respiratory tract deposition of diesel combustion particles in patients with COPD during spontaneous breathing.

**Methods:**

Ten COPD patients and seven healthy subjects inhaled diesel exhaust particles generated during idling and transient driving in an exposure chamber. The respiratory tract deposition of the particles was measured in the size range 10–500 nm during spontaneous breathing.

**Results:**

The deposited dose rate increased with increasing severity of the disease. However, the deposition probability of the ultrafine combustion particles (< 100 nm) was decreased in COPD patients. The deposition probability was associated with both breathing parameters and lung function, but could be predicted only based on lung function.

**Conclusions:**

The higher deposited dose rate of inhaled air pollution particles in COPD patients may be one of the factors contributing to their increased vulnerability. The strong correlations between lung function and particle deposition, especially in the size range of 20–30 nm, suggest that altered particle deposition could be used as an indicator respiratory disease.

## Background

More than 200 million people suffer from chronic obstructive pulmonary disease (COPD) and of these 3 million die each year [1,2]. Today the disease is the fourth most common cause of death globally and it is predicted to become the third within the next decade. One crucial consequence of COPD is an increased susceptibility to air pollution, which could be partly related to a deviation in the uptake of airborne particles during breathing. Air pollution in urban and indoor environments is one of the leading global adverse health factors [3]. Most of these air pollution particles originate from combustion.

The two most important individual characteristics determining the probability of an inhaled particle to deposit in the lungs are breathing pattern and lung morphology. Both these are substantially altered in patients with COPD. Nevertheless, few studies are available on respiratory tract particle deposition in COPD patients in the size range below 1 μm, where much of the ambient airborne particle pollution is found [4-6]. Of these studies, only one investigate a size range which partly covers typical ambient aerosol (20–240 nm, [4]), while the other two examines particles of a single size (33 nm and 100 nm, respectively, [5,6]). Two of these three studies employ controlled pre-determined breathing patterns and are therefore difficult to use for estimation of real-world exposure. Since just one of the studies ([5], using 33 nm particles) varied flow rates while the others kept them constant, relationships between breathing pattern and deposition are not easy to establish.

Thus, several important aspects of the deposition of particles in COPD patients require further examination. The probability of an inhaled particle to deposit in the lungs may vary from more than 80% for particles smaller than 30 nm in diameter to below 20% for particles around 500 nm. For particle sizes other than 33 nm, deposition in COPD during spontaneous breathing is still unclear. Measurements on lung deposition of particles likely to be found in the environment, such as those from combustion sources, have not been performed in COPD.

The objective of this work was to experimentally investigate the respiratory tract deposition in COPD patients for two types of diesel exhaust particles (DEPs) in the range 10 to 500 nm during spontaneous breathing and to seek relationships between deposition, breathing parameters and lung function (static and dynamic spirometry, including diffusion capacity). DEPs were used because they are ubiquitous in the urban atmosphere, have a well-characterized toxicology and are described to cause adverse respiratory and cardiovascular responses [7-10]. It has also been shown that DEPs in the range 10–300 nm have a similar deposition probability for healthy subjects as spherical hydrophobic particles of similar mobility diameter [11]. Epidemiological studies have linked exposure to DEPs with increased COPD mortality [12].

## Results

### Diesel exhaust particles

**Figure 1:**
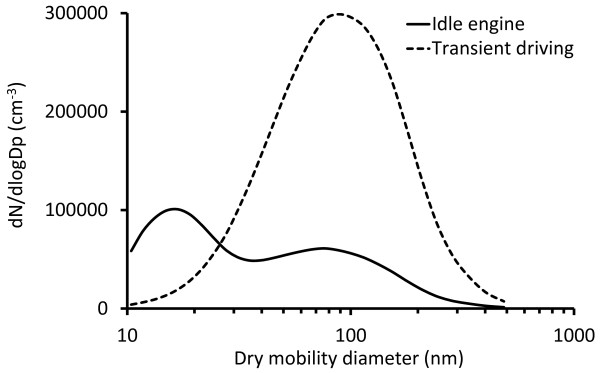
Size distributions of the diesel exhaust particles (DEPs).

The respiratory tract deposition of DEPs from both idling engine and simulated transient driving was investigated (Figure 1). Idling engine emitted a bimodal aerosol consisting of a nucleation mode with geometric mean diameter (GMD) at 16 nm (geometric standard deviation, σ_g_, 1.52) and an accumulation mode with a GMD of 75 nm (σ_g_ 1.98). The particles from transient driving were unimodal with a GMD of 88 nm (σ_g_ 1.97). Number concentrations were 82000 ± 22000 cm^-3^ for idling and 222000 ± 13000 cm^-3^ for transient driving. Further information about the aerosols is provided in the online Additional file 1 and in a previous publication [11].

### Subject characteristics

Subject demographics, breathing parameters and pulmonary function data of the subjects are summarized in Table 1. The subjects with COPD had a significantly lower FEV_1_/FVC, FEV_1_, DL_CO_/VA and PEF than the healthy group. Diffusion capacity of carbon monoxide (DL_CO_) was not obtained from two of the healthy subjects. Some of the healthy subjects breathed with a lower frequency, f, and a higher tidal volume, V_T_, than expected at rest [13]. The spontaneous breathing of the COPD patients was in reasonable agreement with estimated normal values [14]. One healthy subject was excluded because breathing was considered forced (f = 4.5 breaths/min, V_T_ = 2.7 L).

**Table 1:** Subject demographics, pulmonary function data and breathing parameters (Mean ± SD)

	**Healthy (n = 7)**		**COPD (n = 10)**	
		**% predicted**		**% predicted**
Age (yrs)	30 ± 8		67 ± 7^‡^	
Gender	2 M/5 F		7 M/3 F	
Height (cm)	168 ± 9		171 ± 7	
Weight (kg)	63 ± 5		73 ± 10*	
Pack years	0		44 ± 19^‡^	
FEV_1_ (L)	3.71 ± 0.96	107 ± 12	1.80 ± 0.45^‡^	66 ± 16^‡^
FEV_1_/FVC	0.79 ± 0.04	95 ± 4	0.49 ± 0.08^‡^	65 ± 10^‡^
FVC (L)	4.44 ± 1.09	110 ± 10	2.85 ± 0.63^†^	81 ± 18
VC (L)	4.69 ± 1.21	115 ± 14	3.69 ± 0.77	102 ± 22*
TLC (L)	6.13 ± 1.31	109 ± 11	6.9 ± 0.9	112 ± 12
RV (L)	1.40 ± 0.20		3.24 ± 0.58^‡^	
RV/TLC	0.24 ± 0.04		0.47 ± 0.08^‡^	
PEF (L/s)	8.15 ± 1.66	105 ± 7	5.51 ± 1.70^†^	75 ± 20^†^
MEF50 (L/s)	4.02 ± 1.14	85 ± 21	0.78 ± 0.33^‡^	20 ± 8^‡^
MEF25 (L/s)	1.56 ± 0.54		0.17 ± 0.06^‡^	
DL_CO_(SB)^a^	8.46 ± 1.99	92 ± 13	5.14 ±0.64^‡^	62 ± 12^†^
DL_CO_/VA ^a^	1.65 ± 0.06	92 ± 3	0.96 ± 0.19^‡^	70 ± 11^‡^
MV (L/min)	8.4 ± 1.5		10.6 ± 2.9	
f (breaths/min)	10.7 ± 3.6		13.1 ± 3.4	
V_T_ (L)	0.86 ± 0.26		0.86 ± 0.28	

Abbreviations: MEF25/50 = maximum expiratory flow rate at 25% and 50% of VC respectively, DL_CO_(SB) = single-breath diffusion capacity for CO [mmol/min/kPa], DL_CO_/VA = diffusion capacity normalized to lung volume [mmol/min/kPa/L], MV = minute ventilation, f = breathing frequency, V_T_ = tidal volume.

*p < 0.05, ^†^p < 0.01, ^‡^p < 0.001.

^a^ n = 5 for healthy subjects.

### Deposition fraction, DF

The deposition fraction (DF) was calculated from the difference between inhaled and exhaled particle concentrations (see Method section, online Additional file 1 and previous publications [15] for details). Each subject had a similar DF for both types of DEPs, which is expected considering the physical and chemical characteristics of the particles [11]. The similar DFs for both exposures also indicate a repeatable behavior of the subjects combined with stability of the experimental set-up. For this reason, the DFs were averaged for the two types of DEPs in the following analysis, if not stated otherwise.

**Figure 2:**
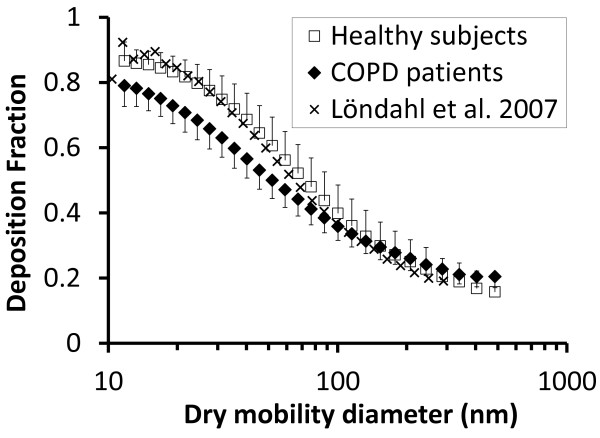
**Measured size-dependent deposition fractions of diesel exhaust particles (DEPs).** Data are shown as group means of both types of DEPs for healthy subjects and COPD patients. For comparison, the deposition fractions are also shown for spherical hydrophobic oil particles for healthy subjects in a previous study (Löndahl et al. 2007). Bars indicate standard deviation between subjects.

For most the ultrafine particles size range (< 100 nm), the DF of inhaled DEPs was significantly lower in COPD patients compared to the healthy group: below 80 nm with p < 0.05 and below 50 nm with p < 0.001 (Figure 2). For particles larger than 100 nm, the DF was equal or higher in the COPD patients, but differences were not significant.

The DF may be calculated not only as the size dependent value, but also as a total DF meaning the fraction of the complete aerosol that is deposited. Unlike the size dependent DF, the total DF is partly determined by the particle size distribution. Table 2 shows values of the total DF by number, surface area and mass of the two types of DEPs. The only significant difference is the total DF of the DEPs by number (p < 0.05). The surface area and mass of the DEPs are dominated by the larger particles (100–500 nm) and for these the COPD patients and the healthy group had a more similar DF.

**Table 2:** Total measured deposited fraction (mean ± SD)

		**Total deposition fraction**		
		**Number**	**Surface area**	**Mass**
Idle	Healthy	0.64 ± 0.06	0.30 ± 0.06	0.27 ± 0.06
	COPD	0.57 ± 0.05*	0.28 ± 0.04	0.29 ± 0.04
Transient	Healthy	0.47 ± 0.08	0.27 ± 0.07	0.27 ± 0.07
	COPD	0.40 ± 0.05*	0.26 ± 0.04	0.26 ± 0.04

*p < 0.05 for COPD compared to healthy group.

### Breathing pattern, lung function and DF

**Figure 3:**
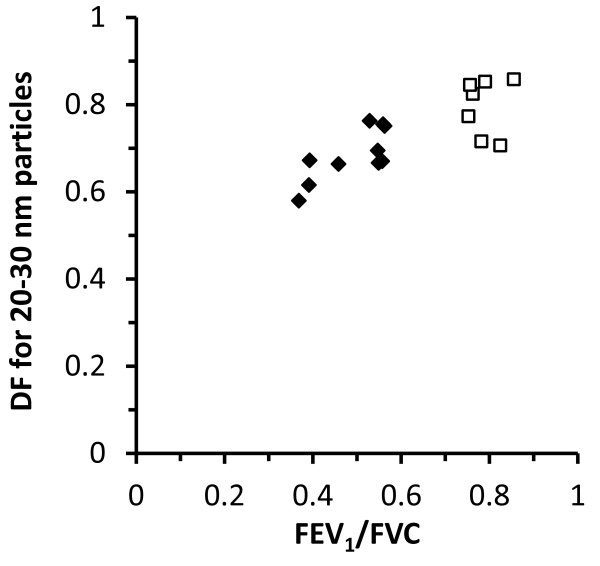
**Deposition fraction (DF) for 20–30 nm particles as function of FEV**_**1**_**/FVC.** Black diamonds (♦) are for COPD patients, white squares (□) for healthy subjects. A similar association between DF and FEV_1_/FVC was found over the complete ultrafine particle size range (< 100 nm).

Although each subject had a similar DF for both types of DEPs, there was considerable variability in DF between the subjects: 0.6-0.9 for 20 nm particles and 0.1-0.2 for 500 nm particles. The variability was related to differences in both breathing pattern and lung function (Table 3 left column). Correlations were typically stronger for smaller sized particles. No significant correlations were found between DF and breathing pattern or lung function for particles larger than 100 nm. The associations shown in Table 3 for 20–30 nm particles were similar for particles in the range 30–100 nm, but typically with a lower level of significance. Notably DF of 20–30 nm particles was strongly positively correlated with lung function deviations characteristic for COPD: FEV_1_ (r = 0.59, p = 0.01), percentage predicted FEV_1_ (r = 0.59, p = 0.01), FEV_1_/FVC (r = 0.79, p = 0.00002), percentage predicted FEV_1_/FVC (r = 0.79, p = 0.0001) and diffusion capacity of carbon monoxide, DL_CO_/VA (r = 0.61, p = 0.02). The correlation between DF and FEV_1_/FVC is illustrated in Figure 3.

**Table 3:** Pearson’s correlation coefficients (r) between subject characteristics and deposition fraction (DF) of 20–30 nm particles of DEPs and deposited dose rate by mass

	**DF 20–30 nm**	**Dose rate (mass)**
FEV_1_	0.59*	−0.26
FEV_1_, % predicted	0.59*	−0.48
FEV_1_/FVC	0.79^‡^	−0.59*
FEV_1_/FVC, % predicted	0.79^‡^	−0.57*
FVC	0.47	−0.03
FVC, % predicted	0.42	−0.40
TLC	−0.32	0.65^†^
RV	−0.57*	0.54*
RV/TLC	−0.50*	0.30
PEF	0.60*	0.006
MEF50	0.58*	−0.46
MEF25	0.61^†^	−0.45
DL_CO_(SB)	0.39	−0.63*
DL_CO_/VA	0.61*	−0.81^‡^
DL_CO_/VA, % predicted	0.48*	−0.70^†^
Pack years	−0.60*	0.42
MV	−0.64^†^	0.77^‡^
f	−0.71^†^	0.28

*p < 0.05, ^†^p < 0.01, ^‡^p < 0.001.

Associations between DF and subject characteristics for all ultrafine particles (< 100 nm) were similar to 20–30 nm particles, but usually with a lower level of significance. No significant correlations were found for particles larger than 100 nm. DF and mass dose are averaged for diesel exhaust particles (DEPs) generated during both idling and transient driving.

COPD patients with emphysema typically have an increased residual volume (RV) as was the case for the participants in this study. There was a clear negative correlation between RV and DF of 20–30 nm particles (r = −0.57, p = 0.02). This indicates that emphysema decreases deposition probability of inhaled ultrafine aerosol particles.

Breathing frequency and minute ventilation, but not tidal volume (V_T_), correlated significantly with DF in the ultrafine particle size interval (Table 3). Increases in breathing frequency and minute ventilation were associated with decrease in DF of ultrafine particles (p < 0.005). No associations between DF and breathing pattern were found for particles larger than 100 nm.

Multivariate linear regression analysis suggested that DF of the ultrafine particles (20–100 nm) for the COPD patients could be predicted based on particle diameter (d_p_, [nm]) and FEV_1_/FVC:

(1) $$\text{DF}\left({\text{d}}_{\text{p}}\right)=0.51\;–0.0047\cdot {\text{d}}_{\text{p}}+0.54\;\cdot {\text{ FEV}}_{1}/\text{FVC}$$

The Pearson’s correlation coefficient (r) for this equation is 0.94.

### Deposited dose rate

**Figure 4:**
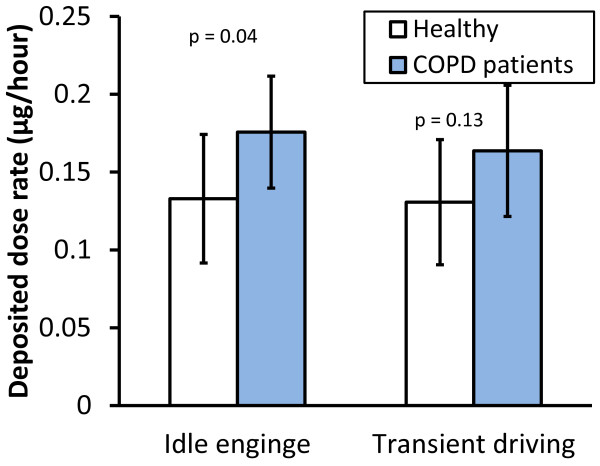
**Deposited particle mass during one hour (i.e. the deposited dose rate) with exposure concentration normalized to 1 μg/m**^**3**^**(mean ± SD)**.

The deposited dose rate here refers to the deposited amount of particles by number, surface area or mass per unit time. The deposited dose rates of DEPs from idle engine and transient driving are provided in Table 4 and illustrated for particle mass in Figure 4. The deposited dose rates are derived from the measured DFs and particle size distribution. For determination of deposited dose rates by surface area and mass also density of the DEPs and TEM image-analysis were used as described in detail by Rissler et al. [11]. Unlike DF, the deposited dose rate differed substantially between the two types of DEPs since it is partly determined by particle size distribution.

**Table 4:** **Total measured deposited dose rate when exposure concentration is normalized to 1 μg/m**^3^

		**Deposited dose per hour (if 1 μg/m**^3^**)**		
		**Number**	**Surface area**	**Mass**
		**(x10**^6^**)**	**(mm**^2^**)**	**(μg)**
Idle	Healthy	695 ± 127	12.3 ± 3.6	0.133 ± 0.041
	COPD	801 ± 178	16.0 ± 3.3*	0.176 ± 0.036*
Transient	Healthy	350 ± 82	19.5 ± 5.9	0.131 ± 0.040
	COPD	386 ± 83	24.5 ± 6.3	0.164 ± 0.042

*p < 0.05 for COPD compared to healthy group.

The mean deposited dose rate tended to be higher for patients with COPD compared to the healthy group, although the difference was only significant for DEPs generated during idling (Figure 4). The reason for the elevated deposited dose rate is that the COPD patients breathed with higher minute ventilation (MV, Table 1) and therefore inhaled more particles. A strong positive correlation was found between MV and deposited mass-dose (r = 0.77, p = 0.0003). However, as previously described, DF was lower in the COPD patients for most particle sizes and this partly counterbalanced the particle uptake.

**Figure 5:**
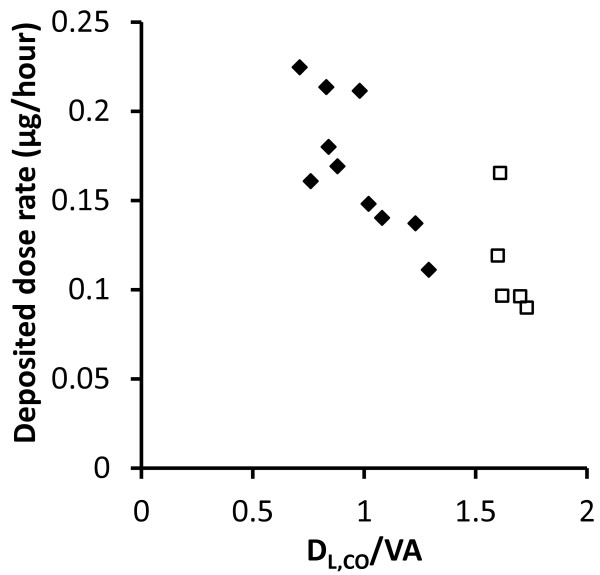
**Deposited dose rate of diesel exhaust particles (DEPs) as function of diffusion capacity of carbon monoxide normalized to lung volume (DL**_**CO**_**/VA).** Black diamonds (♦) are for COPD patients and white squares (□) healthy subjects.

As shown in Table 3 and Figure 5, the deposited mass of DEPs per hour (deposited dose rate by mass) increased with disease severity (i.e. with decrease in FEV_1_, FEV_1_/FVC and DL_CO_/VA). Most significant was the association between deposited dose rate by mass and DL_CO_/VA (p = 0.0004, Figure 5). The inter-subject variability in deposited dose rate was larger than the variability in DF because of substantial variation in minute ventilation. Based on the experimental results, the individual deposited dose rates ranged from 0.09 to 0.22 μg/hour with the particle concentration normalized to 1 μg/m^3^ of DEPs.

## Discussion

The respiratory tract deposition of two types of DEPs was determined in the particle size range 10–500 nm for COPD patients and healthy controls. In contrast to most previous studies, in which subjects breathed according to a pre-determined pattern, spontaneous breathing was used here to resemble real-world exposure. It was found that the respiratory tract deposition of DEPs was closely associated with both breathing parameters and lung function. The deposited dose rate increased with COPD severity, mainly due to increased minute ventilation. The two types of particles studied are representative for DEPs in general as reported in several previous studies [11].

### Deposition fraction, DF

The size-resolved DF of DEPs for the healthy group of subjects (Figure 1) was in agreement with previous measurements for hydrophobic spherical particles [16]. The DF has previously been determined for particles from diesel exhaust, traffic and biomass combustion in healthy subjects [17-19]. It was shown that the DF of combustion particles could be predicted with high accuracy (within 10%) by using the DFs for hydrophobic particles, but with an adjustment for particle size changes due to absorption of water vapor in the lungs [17,18]. The relative humidity in the lungs is high, 99.5%, and therefore inhaled non-hydrophobic particles grow by uptake of water. It is the size of the particles inside the lungs that determines deposition rather than the measured dry size. The DEPs in this study were hydrophobic due to low sulfur content in the fuel [11]. When the deposited dose rate is calculated, density and agglomeration also must be considered [18].

Both images from electron microscopy and density measurements showed that the DEPs were highly agglomerated. Experiments with a cast of human lung airways have suggested that agglomerated particles may have a somewhat higher DF at lung bifurcations than spherical particles with equal mobility diameters [20]. However, the difference in DF between DEPs and hydrophobic spheres of equal mobility size was insignificant for human subjects (Figure 2, see also [11]). The close similarity in DF between DEPs and spherical oil particles is in general agreement with theory. Particles below 300 nm in diameter deposit primarily through Brownian diffusion – the movement caused by random collisions with gas molecules. For hydrophobic particles in this size range, deviations in density and shape have a minor impact on diffusion speed and, consequently, do not affect the DF, provided that particle size is given in mobility diameter, as in this research. The mobility diameter is the commonly used diameter for particles < 0.5 μm. For spherical particles, it is identical to the geometric diameter (i.e. the diameter measured by a ruler on a picture).

### Breathing pattern, lung function and DF

The associations between subject characteristics and DF were in general most significant for particles in the range 20–30 nm, although similar trends were clear over the complete ultrafine size range. The lower DF for ultrafine particles in COPD could partly be explained by variations in breathing pattern. As expected [14], the COPD patients had a higher breathing frequency and minute ventilation than the healthy group (Table 1). This reduces residence time in the lungs, which decreases the DF as less time for diffusion is available.

It is likely that not only alterations in breathing pattern but also variations in airway morphology may reduce the DF in COPD. According to the majority of the lung deposition models, particles in the range 20–70 nm deposit by diffusion, primarily in the alveolar region. In patients with emphysema, the air spaces are enlarged due to destruction of the alveolar walls [21]. As a consequence, the particles have to travel a longer distance before deposition. Hence, the deposition probability decreases. Comparisons with the common ICRP model (International Commission on Radiological Protection) are further elaborated in the online Additional file 1.

Three previous studies have investigated respiratory tract deposition of aerosol particles smaller than 0.5 μm in COPD patients [4-6]. Brown et al. [5] studied the deposition of particles with a count mean diameter of 33 nm (σ_g_ 1.7) during spontaneous breathing in 9 healthy subjects and 10 patients with COPD, whereof 7 were bronchitic and 3 emphysematic (defined as those with percentage predicted DL_CO_/VA < 60%). The COPD patients with emphysema had a significantly lower DF than the healthy group, whereas those classified as bronchitic had a higher. In agreement with the emphysematic group we found a decrease in DF of ultrafine particles.

Comparison of the DF between this study and the two studies in which pre-determined breathing patterns were used is less straightforward. Anderson et al. [4] determined the deposition of hydrophobic 20–240 nm particles in 10 healthy subjects and 8 patients, whereof 5 and 3 had obstructive and restrictive lung disease, respectively. Compared to the healthy group, they found that DF was increased in patients with obstructive lung disease but remained unchanged in those with restrictive lung disease. However, the subjects with obstructive lung disease had difficulties following the pre-determined breathing pattern and this was suggested as a possible explanation to the increased DF. Möller et al. [6] also found a small increase in DF during deep inhalation, but since an 8-second breath hold was carried out at the end of inhalation, results are not comparable.

Several correlations were found between DF and lung function in the present study (Table 3). Brown et al. did not find any associations between DF and lung function across all subjects, but observed relationships between DF and percentage predicted FEV_1_ and percentage predicted DL_CO_/VA in the group of COPD patients alone and in the combined group of bronchitic and healthy subjects. The correlations between DF and lung function in the COPD patients were in close agreement with those obtained here. Möller et al. did not report on relationships between DF and lung function and no associations were demonstrated in the study by Anderson et al.

The strong correlations observed between lung function and the DF of particles in the range 20–30 nm suggest that determination of the DF for particles of this size may be a suitable tool for evaluation of respiratory disease. It is argued above that not only breathing pattern, but also airway morphology is a reason for the abnormal deposition. Numerous studies investigate the use of deposition of aerosol particles as a tool to diagnose lung disease [22-24]. However, these studies focus on particles larger than 400 nm.

### Deposited dose rate

The COPD patients were found to have an increased deposited dose rate of DEPs generated during idling along with a tendency towards a significantly increased deposited dose rate for particles emitted during transient driving (Figure 4). The increased deposited dose rate is in agreement with the results of Brown et al [5]. The reason for the increase in deposited dose rate is the higher minute ventilation in COPD. The subjects with the highest deposited dose rates received about 2.5 times more particles than those with the lowest (Figure 5).

A higher deposited dose rate may be one possible explanation for the increased vulnerability to air pollution exposure found in many toxicological and epidemiological studies in patients with COPD. The deposited dose rate, unlike the size dependent DF, is influenced by size distribution, which varies between different diesel engines. At similar mass concentrations, the number of particles in the air will be larger if the size distribution is dominated by many small particles rather than a few large ones. Therefore, the deposited dose rate of DEPs may vary. When calculating the deposited dose rate for aggregated particles, such as DEPs, the particles effective density must be considered (see online Additional file 1).

## Conclusion

In summary, the COPD patients had an increased deposited dose rate of DEPs, primarily because of heightened minute ventilation (MV). Increased deposited dose rates are believed to enhance the harmful effects of inhaled particles. A strong association was found between severity of COPD and deposited dose rate of inhaled DEPs since low DL_CO_/VA and FEV_1_/FVC resulted in a high respiratory tract deposition. Strong associations were also found between the deposition fraction and lung function, in particular for 20–30 nm particles. The results suggest that measurements of the respiratory tract deposition of 20–30 nm particles may be valuable in the evaluation of respiratory disease.

Since the deposition of DEPs for healthy subjects was similar to the deposition of spherical hydrophobic particles of similar mobility size (as is also expected from theory), the presented results could be assumed to apply not only to DEPs but also to other spherical or agglomerated hydrophobic nanoparticles in at least the range 10–300 nm.

## Methods

### Subjects

Two groups of volunteers were recruited: I) 8 healthy non-smokers with normal lung function (3 men, 5 women, age 23–43 yrs, median 26 yrs), and II) 10 patients with COPD (7 men, 3 women, age 59–78 yrs, median 65 yrs). The COPD patients were ex-smokers with a smoking history of at least 10 pack years and disease severity ranging from stage 2–3, according to the 2008 GOLD guidelines [25]. The study was approved by the local ethics committee and performed in accordance with the Declaration of Helsinki. Informed written consent was obtained from all subjects.

### Aerosol generation

Subjects were exposed to diesel exhaust particles (DEPs) generated by a standard truck engine (Volvo TD40 GJE, 4.0 L, four cylinders, 1996) with no exhaust after-treatment. The engine was operated in a motor test bench in order to simulate two typical engine loads: idling and transient load consistent with the urban driving part of the standardized European Transient Cycle (ETC.) protocol. The concentrated DEPs from the engine exhaust were diluted in a procedure optimized to mimic ambient conditions and thereafter introduced into a human exposure chamber (described in detail elsewhere [7,8]). DEPs from idling and transient load were studied separately.

The daily average mass concentration of DEPs in the chamber was 59 ± 5 μg/m^3^ (as PM_1_) during idling and 300 ± 2 μg/m^3^ during transient load, as measured by a tapered element oscillating microbalance (TEOM). Further information regarding the physical and chemical characterization of the DEPs is provided in the online Additional file 1 and a previous publication [11].

### Respiratory tract deposition

The subjects completed two exposure sessions, one for each type of DEPs. The sessions were randomized on different days and the aerosol type was unknown to the subjects. Each session began with a 3-minute test period for subjects to get accustomed to the equipment, followed by an exposure of 2x15 minutes.

The respiratory tract deposition of the DEPs was determined by a novel method (RESPI) [15]. In summary, subjects wearing a nose clip breathed spontaneously through a mouthpiece while sitting in a relaxed position. The concentration of DEPs in the range 10–500 nm was measured in separate containers for inhaled and exhaled air with a scanning mobility particle sizer (SMPS). Thereafter the deposition fraction (DF) was determined by comparing the size-resolved number of DEPs in the two containers. Corrections were made for particle losses in the instrument and for mouthpiece dead space [15,26]. The DEPs were dried to below 20% relative humidity before measurement. Further information is provided in the online Additional file 1 and in a separate publication [11].

### Calculations

The deposited dose per hour of exposure (the deposited dose rate) was derived as the probability of the DEPs to deposit (DF) multiplied by the inhaled amount of particles by mass or number during one hour. Calculation of the surface area of the deposited particles involves uncertain assumptions on particle structure and is discussed in the online Additional file 1. To facilitate comparisons between the subjects, the concentration of the inhaled DEPs was normalized to 1 μg/m^3^ for the deposited dose rate calculation. The size-dependent mass concentration was determined by combining measurements of particle size distribution (SMPS) and effective density (APM, Aerosol Particle Mass analyzer).

Differences between data means were evaluated with the t-test using SPSS (SPSS Inc., IBM Corporation, version 19). Dependence between variables was investigated with Pearson’s correlation and multivariate linear modeling across all subjects. A p-value of less than 0.05 was considered significant.

## Abbreviations

COPD: Chronic Obstructive Pulmonary Disease; DEPs: Diesel Exhaust Particles; DF, Deposition Fraction; DF20-30: Deposition Fraction of 20–30 nm particles; DLCO: Diffusion Capacity of Carbon monoxide; DLCO/VA: Diffusion Capacity of Carbon monoxide normalized to lung volume; f: breathing frequency; FEV1, Forced Expiratory Volume in one second; FEV: Forced Vital Capacity; MEF25/50: Maximum Expiratory Flow rate at 25% and 50% of VC respectively; MV: Minute Ventilation; PEF: Peak Expiratory Flow; PM: Particulate Matter; TLC: Total Lung Capacity; VC: Vital Capacity; VT: Tidal Volume.

## Competing interests

The authors declare they have no competing financial interests.

## Authors’ contributions

JL, TS, ABl and ES designed the research; JL, ABe and CB performed the experiments; JL, ABe and JR analyzed the data; JL and ABe wrote the paper. All authors contributed significantly to data evaluation and finalization of the text. All authors read and approved the final manuscript.

## Supplementary Material

Additional file 1 Diesel deposition for COPD. Click here for file
