# Supplementary material for: Experimental determination of the respiratory tract deposition of diesel combustion particles in patients with chronic obstructive pulmonary disease
Source: Part Fibre Toxicol. 2012 Jul 28;9:30. doi: 10.1186/1743-8977-9-30 (PMC3464711; doi:10.1186/1743-8977-9-30)
Supplement: Additional file 1 — Diesel deposition for COPD. [file 1743-8977-9-30-S1.doc]

Online supplement, PFT, Diesel deposition for COPD

# Experimental Determination of the Respiratory Tract Deposition of Diesel Combustion Particles in Patients with Chronic Obstructive Pulmonary Disease

*Jakob Löndahl, Erik Swietlicki, Jenny Rissler, Agneta Bengtsson, Christoffer Boman, Anders Blomberg and Thomas Sandström*

**Contents**

1. Characterization of the diesel exhaust particles

2. Comparison with lung deposition models

**1. Characterization of the diesel exhaust particles**

The diesel exhaust particles (DEPs) used in this study have been extensively characterized previously and could be considered typical for engine combustion of a modern diesel fuel [1]. An MK1 diesel fuel with sulphur content ≤10 ppm was used. The residence time of the DEP in the exposure chamber was about 10 minutes.

The set-up of the inhalation system is shown in Figure 1. During exposures the number size distribution of the DEPs was measured with an scanning mobility particle sizer (SMPS), consisting of a condensation particle counter (CPC, model 3010, TSI Inc.) and a differential mobility analyzer (DMA, Vienna type medium, 28 cm long, inner radius 25.0 mm, outer radius 32.4 mm, aerosol flow 0.95 L/min, sheath flow 9.6 L/min). The mass concentration of the DEPs was in addition measured with a tapered element oscillating microbalance (TEOM 1400, Rupprecht&Patashnick Co., Inc., US) and gravimetrically with standard glass fiber filter sampling. The minute ventilation and breathing frequency were measured by a pneumotachograph (Type 2, Dr. Fenyves und Gut, Germany) connected to the container for exhaled air. Flows were corrected to body temperature and pressure saturated (BTPS).

The DEPs were further characterized by the DMA-Aerosol Particles Mass analyzer ([2], APM system, consisting of a DMA coupled in series with the APM, KANOMAX, model 3600). The DMA-APM is an online method, measuring the relationship between the mass of individual particles and their mobility diameter (*d_me_*). This characterization also results in information about the agglomeration state of the particles and enables the estimation of mass size distributions and mass dose, as well as surface area distributions and surface area dose, from the measured number size distributions and lung deposition fractions. DMA-APM measurements were performed for particles with mobility diameters of 50-350 nm. The DMA-APM characterization and surface area calculations are described in more detail by Rissler et al. [1].

An error in the measurements may occur if the inhaled particles change size because of, for example, agglomerate restructuring or evaporation. Therefore particle restructuring was studied separately [1]. The restructuring was negligible (< 3 %) for a humidity and temperature similar to the lungs.

The individual measured deposition fractions are shown in Figure 2.

**2. Comparison with lung deposition models**

Experimental data were compared with theoretical values of the respiratory tract deposition fractions (DF) calculated with the well-established ICRP model [3]. Model estimates were based on each subject’s individual mean breathing frequency (f), mean tidal volume (V_T_) and functional residual capacity (FRC). In this size range the ICRP model is based on a limited amount of experimental data and several later studies have reported deviations from its values (see summary p 29 in [4]). The model does not include any parameters for lung disease.

The measured DF was largely in agreement with the model, but there were some noteworthy discrepancies (Figure 3). For the healthy subjects measured DF was 0.01-0.10 higher than modeled for all sizes. However, this is basically in agreement with the later developed MPPD model (version 2.11; Chemical Industry Institute of Toxicology, Research Triangle Park, NC), which show higher deposition values in this interval than the ICRP model. According to the ICRP model, the increased breathing frequency and V_T_ for the COPD patients would result in an average DF_modelled_ which is 0.02-0.06 lower than for the healthy group for all particle sizes. However, for particles smaller than 70 nm the patients had a DF 0.06-0.10 below the values for healthy subjects, while for particles larger than 200 nm DF was 0.02-0.06 above. This shows that breathing pattern alone could not explain the difference in DF between healthy subjects and COPD patients.

The best lung function predictor for deposited dose rate was the minute ventilation (MV) as illustrated in Figure 4. Regression modeling suggests that the mass dose of DEPs, averaged for idling and transient driving, in µg/hour could be estimated as:

Dose rate (µg/hour) = C · (0.08 + 0.008·”MV”)

where C is the inhaled particle concentration in µg/m^3^ and MV (minute ventilation) is in L/min.

Reference List

1. Rissler J, Swietlicki E, Bengtsson A, Boman C, Pagels J, Sandström T, Blomberg A, Löndahl J: **Experimental determination of deposition of diesel exhaust particles in the human respiratory tract.** *Journal of Aerosol Science* 2012, **Accepted for publication**.

2. Mcmurry PH, Wang X, Park K, Ehara K: **The relationship between mass and mobility for atmospheric particles: A new technique for measuring particle density.** *Aerosol Science and Technology* 2002, **36:**227-238.

3. ICRP Publication 66: *Human Respiratory Tract Model for Radiological Protection, 66.* International Comission on Radiological Protection; 1995.

4. Löndahl J: **Experimental determination of the deposition of aerosol particles in the human respiratory tract.** *PhD Thesis.* Lund University; 2009.

Figure 1. Set-up of the measurement system.

**Figure 2. Individual deposition fractions (DF) for healthy subjects (solid lines) and COPD patients (dotted lines).** Data are given as means for the two types of diesel exhaust particles (DEPs), as each individual had a similar DF for both types of particles.

Figure 3. Measured and modeled deposition fractions for COPD patients and healthy subjects. In the model breathing patterns of individual subjects were used, but no changes were made with respect to lung function.

Figure 4. Deposited dose rate by mass as function of minute ventilation (MV). Black diamonds (♦) are for COPD patients, white squares (□) for healthy subjects.
